# The Advantages and Disadvantages of Integrated Care Implementation in Central and Eastern Europe – Perspective from 9 CEE Countries

**DOI:** 10.5334/ijic.5632

**Published:** 2021-11-08

**Authors:** Donata Kurpas, Dorota Stefanicka-Wojtas, Andrei Shpakou, David Halata, András Mohos, Aelita Skarbaliene, Gindrovel Dumitra, Ludmila Klimatckaia, Jana Bendova, Victoria Tkachenko

**Affiliations:** 1 Wroclaw Medical University, Faculty of Medicine, Wroclaw, Poland; 2 IFIC Board Member; EURIPA; 3 Yanka Kupala State University of Grodno, Grodno, Belarus; 4 VicusMedicus, s.r.o., Hostalkova, Czech Republic; Faculty of Medicine in Hradec Kralove, Charles University, Czech Republic;; 5 EURIPA; 6 University of Szeged, Faculty of Medicine, Family Medicine Department, Hungary; 7 Klaipeda University, Faculty of Health Sciences, Klaipeda, Lithuania; 8 Craiova, University of Medicine and Pharmacy; National Society of Family Medicine, Romania; 9 Krasnoyarsk State Pedagogical University named after V.P. Astafiev, Krasnoyarsk, Russia; 10 BENMEDIKA s.r.o, Senec; Comenius University, Faculty of Medicine, Bratislava, Slovakia; 11 Shupyk National Healthcare University of Ukraine, Kiev, Ukraine

**Keywords:** Central and Eastern Europe, integrated care, implementation, health care, social care

## Abstract

**Introduction:**

Health and social care systems in Central and Eastern European (CEE) countries have undergone significant changes and are currently dealing with serious problems of system disintegration, coordination and a lack of control over the market environment.

**Description:**

The increased health needs related to the ageing society and epidemiological patterns in these countries also require funding needs to increase, rationing to be reformed, sectors to be integrated (the managed care approach), and an analytical information base to be developed if supervision of new technological approaches is to improve. The period of system transitions in CEE countries entailed significant changes in their health systems, including health care financing.

**Discussion:**

Large deficits in the public financing of health systems were just one of the challenges arising from the economic downturn of the 1990s, which was coupled with inflation, increasing unemployment, low salaries, a large informal sector and tax evasion in a number of CEE countries. During the communist period, there was universal access to a wide range of health services, proving it difficult to retain this coverage. As a result, many states sought to ration publicly funded health services – for example, through patient cost-sharing or decreasing the scope of basic benefits. Yet, not all of these reform plans were implemented, and in fact, some were rolled back or not implemented at all due to a lack of social or political consensus.

**Conclusion:**

CEE health systems had come to practice implicit rationing in the form of under-the-table payments from patients, quasi-formal payments to providers to compensate for lack of funding, and long waiting lists forcing patients to the private sector. All these difficulties pose a challenge to the implementation of integrated care.

## Introduction

The WHO Regional Office for Europe defined integrated health service delivery as an approach to strengthen people-centred health systems through the promotion of the comprehensive delivery of quality services across the life-course, designed according to the multidimensional needs of the population and the individual and delivered by a coordinated multidisciplinary team of providers working across settings and levels of care [1]. It should be effectively managed to ensure optimal outcomes and the appropriate use of resources based on the best available evidence, with feedback loops to continuously improve performance and to tackle upstream causes of ill health, and to promote wellbeing through intersectoral and multisectoral actions [2].

Health and social care systems in Central and Eastern Europe (CEE) countries have undergone significant changes and are now facing severe problems related to disintegration, coordination, system and lack of control over the market environment. Increased health needs related to ageing populations and epidemiological patterns in these countries also require increased funding, rationing reform, sector integration (managed care approach), and the development of an analytical information base if the governance of the new technological approach is to be improved. The period of systemic transformations in the CEE countries resulted in significant changes in their health care systems, including health care financing. Large deficits in the public funding of health care systems were just one of the challenges of the economic slowdown of the 1990s, which was accompanied by inflation, rising unemployment, low wages, a large informal sector and tax avoidance in many CEE countries. During the communist era, access to a wide range of health services was common, and maintaining this coverage proved difficult. As a result, many countries sought rationalisation of publicly funded health services – for example, by sharing patient costs or reducing basic services. However, not all of these reform plans were implemented, and some were rolled back (or not implemented in the first place) due to a lack of social or political consensus. Health care systems in CEE also started to use clandestine rationing in the form of hidden payments from patients, quasi-formal payments to health care providers to compensate for lack of funds, and long waiting lists forced patients to move to the private sector. All these difficulties pose a challenge to the implementation of integrated care [3].

## Purpose of the policy paper

The article presents the goals and steps for Central and Eastern European countries (Belarus, Czech Republic, Hungary, Lithuania, Poland, Romania, Russia, Slovakia, Ukraine) in implementing integrated care based on experiences from nine national stakeholders. The key approaches that have been developed in these countries to implement integrated care in practice, the assessment of key barriers and factors facilitating the adoption of integrated care in policy and practice, and common key challenges and opportunities for integrated care are discussed through this policy paper.

## Methodology

The nine countries were selected for the analysis below due to the similarity of history and current assumptions of health and social care systems (this was established in previous joint projects). Nine stakeholders (co-authors of the paper) were invited to analyse national solutions for integrated care. All stakeholders are involved as experts in shaping local and national policies for primary health care. The main method was an analysis of existing articles and national regulations and an individual opinion of the stakeholders on solutions for integrated care in their countries. The national stakeholders chose the most appropriate documents for each country. Through the desk research method, it was possible to extract it in the form of published literature and national documents. Extracted secondary data are discussed in this article.

## Analysis

### Integration between health and social care

In all nine countries, the integration between individual sectors of the health care system is needed. Health and social care are separated at the level of funding and organisation. There are separate ministries of health and social services. Only in **Hungary** and **Romania** can be seen an integration across sectors. In **Romania**, there are two main types of integration. The first is intra-branch (between individual medical organisations, health and social services), and the second is between a medical organisation (a social protection and support body) and a person. In **Hungary**, it is operated by the Ministry of Human Resources – a multifunctional organisation. It is responsible for the functioning of the national health care and welfare system, developing the complete education system, protecting the national cultural heritage, regulating children and youth-related projects, and sports development. Despite the unified top institutional leadership, the individual sectors work separately, and functional harmonisation is not perfect. Since the COVID pandemic, the Ministry of Interior also supervises and organises the protection against the pandemic.

### Change in health care systems

All 9 CEE countries started with a similar hospital base in the Semashko style system. However, the starting point prior to the transformation process was different, and the result is quite varied.

After the transformation in **Belarus**, the health care system is based on government funding (about 65% of health sector expenditure). Segments of insurance and private systems are in relative infancy [4]. In **Hungary**, in the 1990s, the functional privatisation of the family practices, the presence of private health services and the CLIV Act of 1997 on health and some decentralisation intension was observed. At this moment, a new milestone can be found with the criminalisation of out-of-pocket payments [5]. The health care system in **Lithuania** functions differently. Initially, it depended on small, local hospitals. Subsequently, most small hospitals were replaced by larger, consolidated ones, i.e. more efficient ones [6]. In **Poland**, the health care system was based on the principle of social solidarity and universal health insurance. Looking at **Romania**, a significant step forward in health care reform was the social health insurance law (Law 145/1997), which transformed GPs into independent providers, directly contracted for their services by the District Health Insurance Houses (DHIH) [7]. The situation is entirely different in **Russia**, where all residents have access to free medical care through compulsory health insurance [8] (Law of the Russian Federation on Medical Insurance of Citizens in the Russian Federation No. 1499-1 of 28 June 1991). The health care system based on hospitals and an increasing number of outpatients specialists (secondary level specialists) exists in **Slovakia**. A referral letter is required to access most specialists, but this gatekeeping role of GPs is rather formal. Yet another solution was applied in **Ukraine**. The National Health Service of Ukraine (NHSU) was established as a new central executive body. The health care providers became non-commercial communal autonomous establishments and started to receive the funding per capita and medical services according to an agreement with NHSU [9,10].

### The approach of countries to PHC reform

After primary health care (PHC) reform, **Belarus** started transforming PHC to improve the prevention and treatment of non-communicable diseases as part of the comprehensive BELMED project. **In the Czech Republic**, primary care started to be delivered by general practitioners (GPs) and paediatricians. Together they play a vital role in health promotion and prevention (e.g. providing counselling and immunisations) and are often the first contact point in the health system, although there is no formal gatekeeping. Patients may consult specialists directly and generally face few barriers (e.g. no user fees in outpatient settings), explaining the comparatively high number of outpatient consultations (11 contacts per person compared to 7.5 for the EU) [11,12,13,14]. Addressing shortages of primary care doctors and regional disparities is a key challenge. Therefore, integrated care is getting more and more attention [15,16,17]. The typical approach taken by **Hungary** in the last few years involves prevention, public health perspective, and the services close to the community came into view. GP clusters were the means of implementation in pilot programmes [18]. In **Lithuania**, however, the government sought to promote the joint provision of health care and social services in the form of orders. Nevertheless, formal support services were slow to develop. The joint provision of social services and health care lacked the active involvement of health professionals, and cooperation often was refused. The 2011–2015 Lithuanian Health System Development Framework and the 2014–2025 Lithuanian Health Programme were the first official documents that described the development and implementation path of the integrated health care services in the country. Strategic direction aimed at reorienting health care systems, prioritising disease prevention, integrated health care and social services, and ensuring continuity of care, promoting coordinated care and case/disease management in all levels of care was proposed [19]. Like many other former eastern bloc countries, **Poland** inherited a poorly arranged PHC system, with too much focus on the treatment of common conditions and relatively low importance given to prophylactic activities. Efforts were made to improve the role and quality of PHC that at that time was a trend visible in many other CEE countries. Specialisation in family medicine was introduced, however to date, there is no clear governmental strategy for PHC [20]. The reform in primary care was introduced in **Romania**, and the old concept based on former medical dispensaries with complete primary care teams was abandoned. The staff was taken over by the new individual medical offices coordinated by the family doctor. Unfortunately, funding was dramatically reduced, and the old teams were limited to a doctor and a nurse. In **Russia**, the PHC system is the basis of the medical care system. It includes measures for prevention, diagnosis, treatment of diseases and conditions, medical rehabilitation, monitoring the course of pregnancy, formation of a healthy lifestyle and hygienic education of the population. Primary health care is organised to bring the medical services to population closer to their place of residence, place of work or study, according to a territorial-participant principle which provides for the formation of groups of the population being served at certain organisations according to their place of residence, place of work or study. Primary pre-hospital medical care is provided by paramedical workers (feldshers, midwives, etc.). Primary care is provided by general practitioners, paediatricians, general paediatricians and/or family doctors). The situation in **Slovakia** is very similar. Formal efforts were made to improve the role and quality of PHC, but the role of PHC is still weak. Although the access to PHC and its continuity in Slovakia is relatively strong, the comprehensiveness of primary care was rated the lowest out of 31 European countries. [21,22,23,24,25] GPs in the Slovak Republic consistently resolve only around 70 per cent of consultations without referral to other specialists, compared to an international benchmark of over 90 per cent. To date, there is no clear governmental strategy for PHC. Whereas in **Ukraine**, three underlying principles were accepted for the future health system development: people-centred, outcomes-oriented and implementation-focused. The following key approaches guide the health system in all its activities: a guaranteed package of services available to all – following the national standards of excellence and professionalism, patient empowerment, collaboration across organisational boundaries in the interest of patients, communities and the wider population, value for money and the most effective, fair and sustainable use of limited resources, accountability to the public, communities and patients that it serves [26].

### The individual role of integrated care and the specific country experience with it

The fragmented nature of today’s health systems in nine CEE countries means that they are becoming increasingly unable to respond to the demands placed upon them. The focus on hospital-based, disease-based and self-contained “silo” curative care models undermines the ability of health systems to provide universal, equitable, high-quality and financially sustainable care. This strategy calls for reforms to reorient health systems and services, shifting away from fragmented supply-oriented models towards health services that put people and communities at their centre to provide them with responsive services coordinated both within and beyond the health sector, irrespective of a country setting and development status).

The Belarusian model of the health and social assistance system is currently in the early stages of formation. For an effective integrated care system in **Belarus**, it is necessary to amend legislation, concretise state concept, financing, compensate for outpatient care, provide 100% access to central heating (gas/electricity), water supply, sewerage, reorient hospital beds and outpatient care. In the **Czech Republic**, the present approach of the state authorities is based on hospital-based care models with marginalising the role of PHC. The **Hungarian** health care system could be described as mostly “disease-centred” and not prevention-focused. Projects for implementing integrated care were launched in **Lithuania** [27]. However, the main barrier to the successful implementation of integrated care is still the lack of local experience. In **Poland** and **Romania**, the focus is placed on hospital-based, disease-based and curative care models. As a result, their health systems are unable to provide universal, equitable, high-quality and financially sustainable care. Meanwhile, **Russia** started the primary health care reform, which was delayed by six months due to COVID-19. The attention is centred on ensuring 100% accessibility for the population to medical services. All models of organisation of primary health care are patient-centred. In order to provide primary health care to residents of settlements located at a considerable distance from the medical organisation and (or) with poor transport accessibility, taking into account climatic and geographical conditions, mobile medical complexes are required [28]. **Slovakia** has no experience with integrated care. The Ministry of Health started to prepare reform aiming to integrate the health and social care and possibly merge two separated ministries – the Ministry of Health and the Ministry of Social Services. However, due to the pandemic, the legislative efforts were halted. WHO and the Ministry of Health of **Ukraine** implemented the list of national clinical guidelines in integrated care for children, non-communicable diseases, mental and behavioural disorders, substance abuse, etc. The cascade of training courses for PHC teams in integrated care of patients with mentioned above disorders was provided in the country. The results concerning the efficacy of these innovations were described in the report on the findings of a nationally representative study on the prevalence of major behavioural and biological risk factors for non-communicable diseases. Additionally, the foundation of the National Health Service of Ukraine (NHSU) and the new principle of financing of PHC centres and the rule fo “money follows the patient” per capita and for medical services according to an agreement with NHSU stimulated concentration on patient-oriented care approach [29].

### The specific integrated activities in the nine CEE countries

In the last ten years, to advance integrated care and plan the integrated activity to cope with the fragmentation problem **Ukraine** and **Lithuania** started to be more comprehensive and focused on integration. In **Belarus**, it can be observed as a joint project of the Red Cross and the “Names” Fund – “Patronage Service in the Regions”, the system of providing medical and social assistance. Also, a new form of social service has been established – social services for foster families. Primary care reform in **the Czech Republic** gave more attention to the integrated care, empowerment of the social, community and long-lasting care provided under PMC. It led to an amended set of suitable quality criteria for GP practices and bonuses mechanisms of reimbursement for those GP practices which reach the desired quality level of the care for patients [30,31,32]. In the last decade in **Hungary**, we could observe duality related to primary care. The first challenge is to sustain the system, and the second is to transform and develop it. Accessible patient data are crucial in high-quality patient care. Currently, almost every provider is connected, most of the patient data are uploaded to the system, and the e-referral and e-prescription system was introduced as well [33,34]. Before regaining independence, institutional care was the only form of social service in **Lithuania**. The situation began to change in 1996 with the adoption of the Law on Social Services, which delegated the development of services to municipalities. The government sought to promote the joint provision of health care and social services in the form of orders. Nevertheless, formal support services are slow to develop. The joint provision of social services and health care lacks the active involvement of health professionals, and cooperation is often refused. Strategic direction aimed at reorienting health care systems, prioritising disease prevention, integrated health care and social services, and ensuring continuity of care, promoting coordinated care and case/ disease management in all levels of care was proposed. **Poland** introduced a hospital network initiative to support the integration of outpatient and inpatient care. Legislation strengthened primary care coordination by introducing multidisciplinary primary care teams to coordinate care pathways, including post-hospital treatment and rehabilitation. Coordination of care will also cover activities in the areas of health promotion and prevention. Both these goals have been recently recognised at the EU level. A community health care system was developed in Romania, but it did not reach satisfactory territorial coverage. Meanwhile, **Russia** decided to reduce the number of medical organisations due to their merger and the incorporation of polyclinics into hospitals. Another trend is an increase in the average size of hospitals. This figure is now markedly higher than in Western countries with large populations. The formation of large medical associations, which include almost all territorial medical services, can also be observed. Unfortunately, in Slovakia, there was no policy development in the last ten years to advance integrated care. The government of **Ukraine** [35] initiated a massive reform of its entire health system to move towards universal health coverage (UHC) and improve the health outcomes of the population. A comprehensive reform strategy was put forward covering four key areas, including 1) health service and delivery, 2) health financing, 3) quality governance of the sector and 4) ensuring essential health system inputs. To improve access to essential medicines, vaccines and diagnostics, the government provides centralised public procurements through transparent mechanisms. However, overall challenges are still related to slow economic growth, rising health care costs partly due to changing epidemiological patterns, deteriorating infrastructure, governance practices and inefficient institutions and an outdated health information system.

### Strengths and weaknesses of integrated activities and how they respond to the needs of the country/population

The most important strength of integrated activities in **Hungary** is the importance of prevention and the intention to provide the services locally. Projects in this field were funded not only from the national budget but also from international funds, mostly EU. Gaps in the coordination of health services are among the biggest problems in **Lithuania** – it is on the list of EU countries with high rates of potentially amenable deaths. However, life expectancy at birth is increasing every year. Lithuania has a considerably higher number of physicians than the EU average and a slightly lower number of nurses than the EU average. The geographic spread of doctors in the country is yet very uneven, i.e. the vast majority (over 71%) of doctors work in the cities. In comparison, 60% of health care needs are provided in the surrounding districts [36,37,38,39,40]. Poland’s record on mortality from treatable conditions is relatively good among EU countries with similar or higher levels of expenditure on health. Yet, the mortality rate is still high and well above the EU average. There are very significant inequalities in life expectancy by education and gender, with men with the lowest level of education living about 12 years shorter than that of the better-educated individuals [3]. The number of doctors is the lowest in the EU; this is also true of nurses. The fraction of general practitioners (GPs) is the second-lowest in the EU [41]. These weaknesses in outpatient care and shortages in the health workforce lead to long waiting times and go partway in explaining why certain indicators, such as unmet health care needs, are worse in Poland than in countries with similar levels of health spending [41]. In **Romania**, the medical sector experiences the difficulties faced by the elderly. The development of an integrated care system is perceived as important because it would determine a greater efficiency of health care. The system weaknesses include the extended territorial coverage with community nurses and health mediators and the ageing of primary care professionals. Integration in **Russian** health care can be divided into two main types. The first one is intrasectoral (between individual medical organisations, health and social services), and the second one is between a medical organisation (social protection, support body) and a person. The state policy of restructuring the hospital health care sector focusing on reducing the volume of inpatient care and transferring some patients to the outpatient stage plays a decisive role. The main positive result of concentration is increased manoeuvrability of resources. Another positive outcome of the concentration is increased access of the population to expensive diagnostic services and the most scarce specialists of polyclinics. The downside of this process is the more complicated logistics of patient traffic. The positive effects of concentration include the possibility to reduce administrative costs. In a simple merger of medical organisations, the main thing is missing – the focus of different medical services to work more closely together [42,43]. The global trend of an ageing population, increasing life expectancy and decreasing birth rates can also be found in **Belarus**. The main problems and threats facing society in the process of ageing of citizens are associated with a decrease in the number of physically fit population, an increase in the demographic burden, a decrease in the financial sustainability of the pension system, an increase in government spending on health and social affairs, a reduction in the supply of qualified personnel to the labour market and a break in intergenerational ties. Primary care is not as effective as it could be in the **Slovak Republic**. The Slovak Republic has a higher amenable mortality rate and acute hospital care than the European Union (EU) average. The GP workforce is also ageing and faces difficulty in recruiting newly qualified doctors. Restrictions on the competencies of GPs are considered a particular obstacle to the potential efficiency gains of stronger primary care. The health system in the Slovak Republic fails to address the growing burden of non-communicable diseases. Healthy life years, a measure of the remaining years that a person of a certain age is expected to live without disability, is extremely low in the Slovak Republic compared to neighbouring and regional comparators. The comprehensiveness of primary care in the Slovak Republic barely changed or even declined in the last 20 years. The Slovak Republic is one of only three countries that reduced the disease management capacity of its GPs in the face of an ageing population. The exception is preventive activities, five of which GPs consider a greater part of their usual practice. However, the small stock of GPs in the Slovak Republic is a key barrier to integrated care. The strengths of integrated activities in **Ukraine** include, for instance, the implementation of new principles of financing the health system. Each patient signed the contract with the primary care doctor. Implementation of the Health Care Guarantee Programme and the Affordable Medicines Reimbursement Programme allows patients to get prescription drugs for their cardiovascular conditions, type 2 diabetes and bronchial asthma free of charge or with a small co-payment [44]. Mental health and tuberculosis services received new regulations. Digital transformation in health care started its development. In addition, the health care workers payment was raised in some settings. The weaknesses of integrated activities include the lack of doctors, especially in the countryside. The patient-oriented system is progressing slowly and financing of health care is still insufficient.

### Key programmes and/or practice approaches to implement integrated care policies

The concept of integrated medical and social assistance was first introduced in **Belarus** in the draft of the revised law on health care. It proposed developing a comprehensive, personalised and integrated approach to the provision of medical care based on the identification of the needs of older citizens, including geriatric services as a unified system of long-term medical care, expanding the practice of organising and supporting schools of “active ageing”, “long-term care”, as well as creating other models of motivation for active longevity of seniors [45].

Primary care reform in the **Czech Republic** gave more attention to the integrated care, empowerment of the social, community and long-lasting care provided under PMC.

**Hungary** has the earlier mentioned Swiss-Hungarian Cooperation Programme to establish GP clusters. In the Human Resources Development Operational Programme (HRDOP) framework, more than 60 GP clusters were established starting from 2017. The Professional Methodological Development of the Health Care System project was responsible for analysing and evaluating the operation of the GP clusters. Based on these experiences, they made recommendations and provided advice on good practices. Oncology and palliative care are some of the leading specialities in this field. The Hungarian OnkoNetwork Programme and Palliative Care Consult Service as part of the international SELFIE (Sustainable Integrated Chronic Care Models for Multi-Morbidity: Delivery, FInancing and Performance) Project is a Horizon 2020 funded EU project that examined integrated care in the field of oncology and palliative care [46].

Several pilot projects for implementing integrated care were launched in **Lithuania**. In July 2012, the Integrated Care Programme started with establishing 70 integrated care teams (social workers, nurses, assistants and physiotherapists) in 21 (out of 60) municipalities. The programme aimed to develop a care system for persons with a long-term chronic illness, integrating social care and nursing, to enable informal caregivers to rest or look for employment. The project exceeded the expectations by serving 1,172 patients and 1,005 family members in 2015 and showed the need for agents who support participatory service development. Moreover, innovators can be successful in reforming practices and making integrated health care real by understanding key dimensions of innovation, strategically using external support and modelling partnerships across levels of bureaucracy. After the qualitative and quantitative evaluations, the Integrated Care Programme was upscaled for implementation in all municipalities of Lithuania by 2016. Another action on implementing good practices for chronic diseases (CHRODIS PLUS) addressed chronic diseases through cross-national initiatives to reduce the burden of chronic diseases while assuring health system sustainability and responsiveness. However, the project revealed the lack of human resources and the need for additional training. The projects provided volunteering as a means of addressing the lack of human resources in care teams. In addition, educational interventions addressed misconceptions and stigmas. Finally, all projects demonstrated the benefits of integrated care. However, the main barrier to their implementation is the lack of local experience [47,48,49].

In **Poland**, the public payers are solely accountable for securing and organising access to health care services and responsible for implementing the Primary Health Care PLUS (PHC PLUS) project to introduce a PHC centred model based on coordinated, proactive and preventive methods relevant to patients’ needs. The National Health Found currently implements the above project. Objectives of the PHC PLUS include improving the quality of medical services at the PHC level, increasing the number of medical services delivered at the PHC level instead of specialist and inpatient care, focusing on prevention rather than reaction and coordination of medical services at the PHC level [50]. In addition, periodic health examinations of adults were recommended for implementation in a model of coordinative care for PHC, which the World Bank suggested [39]. This patient-oriented strategy appears better adapted to the current health care environment and demographic trends [3,50,51,52].

Another important initiative in Poland is the Regions4PerMed Project (funded from the European Union’s Horizon 2020 research and innovation programme), whose overarching goal is to set up the first interregional cooperation on PM, align strategies and financial instruments, identify key investment areas and release a European regional agenda to foster the delivery of PH (personalised health) services to patients and citizens. Overall, this will result in a coherent, science-founded basis for decision-making [53,54].

In **Romania**, the medical-social units were established, which address the care of patients with social problems and chronic diseases. However, they do not cover the entire country territory.

A national policy of integrated medical and social assistance exists in **Russia**. The main document of the national policy in relation to integrated medical and social assistance is the “Older Generation” project within the framework of the “Demography” national project [55] developed on the basis of the “Health Development Strategy” [56] and “Action Strategy for the Benefits of Older Citizens” [57]. The project is connected with the state programmes “Social support of citizens” [58] and “Development of Health Care” [59]. The “Old Age in Joy” Foundation acts as an expert and developer of the methodology [60]. Within the framework of implementing an integrated system of medical and social assistance and care for elderly citizens and disabled people, a balanced social service is provided in a semi-stationary, stationary form and at home with the involvement of the patronage service and nurses. Support for family care is under development. The procedure for interaction between medical organisations and social service organisations was established, including the synchronisation of information systems and methods of transferring information about the patient’s condition to their relatives and social service organisations. Thanks to the financial support of the project, modern hospital-replacing technologies were developed, which make it possible to compensate for the lack of care from a relative and ensure that citizens live in a familiar environment. Regional geriatric centres and gerontological departments function in 70 of 85 constituent entities of Russia. A set of measures was introduced to prevent falls and fractures and facilitate the early detection of cognitive impairments. In the regions, there is a “single coordination centre” to coordinate the long-term care system. All conditions were designed to allow non-governmental organisations to operate in the market [61]. However, actual problems include low coordination of actions (interagency) and participants, difficulty establishing care services in which health care facilities and services should be involved and the need for a well-coordinated system in the country.

In **Ukraine**, key programmes and practice approaches to implement integrated care policies are the Health Care Guarantee Programme and the Affordable Medicines Reimbursement Programme. The key focus of the 2021 Health Guarantee Programme is bringing health care closer to the patient through the development of outpatient care and integrated health services.

Unfortunately, there are no programmes and/or practice approaches to implement integrated care policies in **Slovakia**.

### Evidence on outcomes of the integrated care policies

The ‘Quadruple Aim’ is centred around overarching goals: improving the individual experience of care; improving the health of populations; reducing the per capita cost of healthcare; and improving the experience of providing care [62].

In the **Czech Republic, Poland** and **Belarus**, the integrated care policies were implemented not long ago, and there is still no evaluation of outcomes. Similarly in **Hungary**, the comprehensive analysis and assessment of these projects are still ongoing, and there are still no final results [18,63]. In **Romania** and **Slovakia**, there is no plan concerning integrated care. Integrated care pilot projects were introduced in 21 **Lithuanian** municipalities in 2012. Burnout of caregivers was reduced by decreasing physical overload and providing possibilities for respite. Cooperative work increased the quality of nurses‘ and carers‘ knowledge and skills, especially concerning bedsores. Integrated care mediation between carers and patients was new and valued. Attained autonomy in the integrated team-based home care pilot projects facilitated the change of the nurse role [64,65]. The “Lean Medicine – Careful Attitude towards Medical Personnel” programme was implemented in **Russia**. Its main objective was improving the availability and quality of medical care for the population by optimising processes and eliminating losses. Directions and pilot projects in Russia concerned redistribution of workload between doctors and nurses, optimisation of outpatient clinic internal logistics, segregation of patient flows, transition to electronic document management, reducing paperwork, open registrar and the new outlook on the outpatient clinic and organisation of medical examinations and health examinations on the principle of a continuous flow of patients following the standards of reception time per patient. The results in pilot polyclinics included increasing the doctors’ work time directly with patients by a factor of 2, reducing the time needed to make an appointment by a factor of 5, reducing queues by up to 8 times and the waiting time for appointments by 12 times, comfortable and accessible environment for patients in outpatient clinics and reducing the time required for medical check-ups and preventive examinations of children [66]. The STEPS survey collected in **Ukraine** provided a wealth of information on NCDs and their associated risk factors, providing, for the first time, comprehensive, internationally comparable and nationally representative data on these diseases and their risk factors in Ukraine and its integrated care in recent years. In addition, the evaluation of studies on the Tb-HIV integrated care and opioid agonist therapy integration into primary care showed positive results and improved outcomes in both patients and clinicians [67,68].

### Advantages and disadvantages of integrated care and key barriers and facilitators to the adoption of integrated care policies in the nine cee countries

In **Belarus, Poland, Czech Republic, Hungary, Russia and Slovakia**, the main approach is patient-centred care focused on health promotion and disease prevention. Advantages of this approach in **Belarus** include faster access to treatment in one place for patients and first signs of multidisciplinary care. In contrast, disadvantages include lack of professional medical staff, insufficient information on integrated programmes and increased workload. In addition, this approach expands existing contacts between PHC and health care. The main barriers in **Belarus** and **Poland** constitute the lack of skills in primary care, staff, links with the local community, monitoring of care quality and performance and understanding of the culture of primary care. Other critical issues are inadequate IT infrastructure, difficulty accessing information from secondary care and general practices working alone [69].

Strong points of the approach in the **Czech Republic** concern mainly care provision at the time and at the place of needs, holistic approach, medical health centre development (group practices), restrictions of the overdiagnosis, health literacy, health economics, telemedicine development, improving care quality and monitoring quality. Weak points include necessary central support (Ministry of Health, insurance companies) and lack of medical staff. Facilitators of such an approach are additional financial and staff resources and already existing contracts between PHC and secondary care. The main barriers are a misunderstanding of PHC role by politicians at national levels and insurance companies, lack of staff, digitalisation of the health care system and telemedicine systems.

Whereas in **Hungary**, the main advantages are faster access to care in one place for patients, multidisciplinary care, patient stratification systems, emphasis on health promotion and disease prevention, monitoring care quality and performance. This approach shows a lack of sufficient human resources (family doctors, primary care nurses and other health care professionals) and funding. The main enablers include the positive attitude of health care workers and patients for the innovations but lack of sufficient human resources (family doctors, primary care nurses and other health care professionals) and financial and infrastructural conditions still can be observed.

Meanwhile, in **Poland**, noticeable advantages are faster access to care in one place for patients, multidisciplinary care, patient stratification systems, emphasis on health promotion and disease prevention and monitoring care quality and performance. On the other hand, the most important disadvantages are lack of medical staff, interest in integrated care programmes on the part of small centres, an increase in the number of procedures performed in a given centre and increased workload. Key enablers are the attitude of politicians at national levels, additional financial and staff resources and already existing contracts between PHC and secondary care.

For this approach in **Slovakia**, the main weak points are the lack of (or ageing) medical staff and residency programmes failing to supply enough staff (not running effectively). At the same time, there is interest declared by the government to take the direction of integrated care, but there is no action. The future is not bright due to the unstable political situation and no legislative efforts.

On the other hand, **Russia** has faster access to care in one place for patients, multidisciplinary care, patient stratification systems, emphasis on health promotion and disease prevention, monitoring care quality and performance. However, the shortage of medical staff and increased workload can be observed. Moreover, citizens’ attitudes towards their health and medical care are not very good. The main facilitators in Russia are the attitude of politicians at national levels, additional financial and staff resources, development of modern hospital-substituting technologies, already existing contracts between PHC and secondary care and also the organisation of conferences and practices for the exchange of experience between regions in Russia and partnership with other countries, including Israel, Poland and Germany. Unfortunately, there are weaknesses concerning real integration processes, poor IT infrastructure and shortages of medical personnel.

The main approach in **Romania** is prevention and supplementing health care workers to primary care. The advantage of this approach is the first contact with patients in the proper setting, but there are also disadvantages like lack of medical staff. Its perception of utility by the media and politicians is an apparent facilitator, but there are problems with human resources financing.

In **Lithuania**, one can observe a comprehensive patient-centred holistic approach, but its main disadvantages include the lack of medical staff and evidence on integrated care. In addition, of course, there is municipal support in service development and participatory involvement of the unit, but organisational issues related to the interaction between team members and administration, tensions due to status differences between social and health care providers, task distributions between team members, lack of teamwork experience and sense of shared responsibility [70].

**Ukraine** introduced new financing principles of the health system (per capita), national protocols in integrated care of some conditions and digital transformation in health care. Each patient signed a contract with a primary care doctor, and now patients get prescription drugs for some conditions free of charge. Mental health and tuberculosis services received new regulations; the health care workers payment was raised in some settings. Regrettably, lack of doctors, especially in the countryside, is a considerable problem that increases workload. Also, the financing of health care is still insufficient. The Cabinet of Ministers of Ukraine, WHO, Ministry of Health of Ukraine, NHSU and local administration organs attempt to change the system, but they face a lack of staff, monitoring of care quality and performance and difficulty to access information from secondary care [71].

### Learning for other countries

In **Belarus**, the conference with international participation, “Current issues in the organisation of coordinated care at home” in late 2019 proved influential in introducing national strategies [72]. In 2018, primary care reform was implemented in the **Czech Republic** taking into account the need for integration as the basis for the integration of the entire health care system. On the other hand, in **Romania**, it is still necessary to introduce integrated strategies and financing. Also, **Slovakia** is a poor example in this field that should not be followed by other countries. Pilot projects implemented in **Lithuania** revealed the importance and principles of building integrated teams. Since the shortage of medical staff is the problem in many countries, efficient teams are essential for implementing integrated care. In **Ukraine**, it is already known that all resources necessary for implementing integrated care have to be specified in detail, the personnel have to be trained and motivated, the financing has to be adequate to cover planned innovations expenses, including the self-cost plus other relevant expenses. Looking at the example of reform in **Poland**, the lesson for other countries is that solutions at the government level supported by the strong position of the payer of medical services should be adequate to the current resources of primary and specialist care were introduced. On the example of **Hungary**, it is evident from international standards that political willingness, appropriate human resources and infrastructural background are crucial for the effective implementation of complex integrated care programmes. In **Russia**, the ongoing health care and social work reforms started from the integration of public health and primary care, including financial.

### Five strategies for integrated people-centred health services (WHO IPCHS framework)

#### Engaging and empowering people and communities

Engaging people in **Belarus** is still planned; right now, there are only local projects. A health literacy centre was established in **the Czech Republic** in 2018, but outcomes are not available yet. Complex intersectoral issues can be seen in **Hungary**. The education system also plays a significant role in people health awareness. National health promotion campaigns, commercials and local targeted initiatives could be successful. Currently, there are only plans and some pilot projects. In **Lithuania**, engaging people is set one of the strategic goals of the Lithuanian Health Strategy 2014–2025. Such projects in **Poland, Romania** and **Slovakia** are still under construction, or only pilots exist. There are local pilot projects and implementation plans for five strategies for integrated people-centred health services (WHO IPCHS framework) in **Russia**. The Health Care Guarantee Programme and the Affordable Medicines Reimbursement Programme that make health care accessible for low-income people to guarantee universal access to services are implemented in **Ukraine**.

#### Strengthening governance and accountability

Initiatives on coordinated care find support mainly in large medical centres with already existing integration processes at different levels in **Belarusian** and **Russian** primary care. In **the Czech Republic**, the policy-makers are the main block against the development of integrated care models. In H**ungary, Lithuania** and **Poland**, a well-functioning health care system is a strategic point for the governments. There is a strong political will to improve it with centralised, absolutely controlled and directed methods, and there is a dialogue at a municipality level. In Poland, integrated care initiatives also find supporters mainly in large medical centres with already existing horizontal and vertical integration processes. While in **Romania**, a strong collaboration between NGOs and political factors in partnership with professional bodies is necessary. In **Ukraine**, the implementation of e-health, electronic medical records, electronic registry of the patients, ICPC-2 classification, e-receipt, and e-sick lists allowed the system to became transparent in decision-making accountability, but one defect left is the absence of a unified approach to medical data collection for further statistical analysis.

#### Reorienting the model of care

In Eastern Europe, there is a need to reorient the model of care so that efficient and effective health care services are designed, purchased and provided through innovative models of care that prioritise primary and community care services and the co-production of health. In **Belarus, Poland** and **Russia**, in reorienting the model of care, the obstacle is the very small number of medical personnel. In **Poland**, the care system also requires a change into primary care based in local communities. In **Russia**, there is also a problem of training professionals in gerontology. The care system in the **Czech Republic** requires a change into primary care based in local communities. The obstacle is the very low support by the government. The **Hungarian** health care system goes through a high-level centralisation nowadays. Therefore, primary care and public health is a prioritised sector in the structural changes. The **Lithuanian** health system is pretty well designed and institutionally stable. However, service delivery continues to be dominated by large and mostly public hospitals, but outpatient service delivery is increasingly mixed. Specialist outpatient care is delivered through the outpatient departments of hospitals or polyclinics and private providers. In addition, private providers play an increasing role. Primary care is provided in either municipality-owned facilities or typically smaller private practices. **Lithuania** has more physicians and fewer nurses per capita than the OECD average [73]. In **Romania**, an attempt was made to reverse the spending pyramid in the health system by reducing hospital spending and developing the primary care sector – unfortunately, unsuccessfully. On the other hand, reorienting the model of care has already started in **Ukraine** with priority on integrated primary care with multidisciplinary and intersectoral cooperation – with secondary and tertiary professionals, public health care specialists, emergence care, social workers, etc.

#### Coordinating services within and across sectors

The pilots for the integrated care initiative in **Poland, Russia and Belarus** have only just started. Their evaluation has not yet been completed. In these countries, more emphasis should be placed on a comprehensive community-based approach to health care with prevention and health promotion as key components and health districts as fundamental units in its implementation; addressing funding disparities between curative and public health interventions; better services for marginalised populations; actions for change management and role of family members in providing health care and training for them. Coordinating services within and across sectors has to be improved in **Ukraine** because primary, secondary care, public health, and social care are separate services with different financial flows. In contrast to previous examples, a multi-level coordination system could fast and well react to the local and general challenges in **Hungary**. In the GP clusters, the leading GP and the public health coordinator are responsible for coordinating the daily work and data collection. Central working groups analyse and evaluate these data and give feedback and suggestions. And the top level is responsible for the integration and coordination of primary and secondary care. In **Romania**, the role of the family doctor as the primary care coordinator should be increased. Eastern Lithuanian Cardiology Programme was set out as a pilot project to transform the delivery of cardiology services in **Lithuania**. Boosting the role of primary care and emphasising the coordination of services proved to be key in reducing the need for hospital outpatient consultations and admissions. A stronger referral system improved the flow of patients among primary care settings, regional hospitals and central and tertiary facilities and training helped to shift the provision of cardiovascular health services to regional hospitals and local clinics. Lithuania’s example showed how an integrated, people-centred way of delivering health services significantly benefits patients and improves the efficiency of the health system [74].

#### Creating an enabling environment

For the four previous strategies to become an operational reality, it is necessary to create an enabling environment that brings together the different stakeholders to undertake transformational change. This is a complex task involving a diverse set of processes to bring about the necessary adjustments in legislative frameworks, financial arrangements and incentives and the reorientation of the workforce and public policy-making. For example, in **the Czech Republic, Romania, Russia, Poland, Slovakia** and **Belarus**, there are no systemic solutions on a national scale. On the other hand, strong methodology support of the National Health Care Service Centre covers a wide area of health management in **Hungary**: human resource and capacity management, quality assurance, continuous improvement of operational efficiency, centralised public procurement, health care and institutional performance appraisal. In **Ukraine** and **Lithuania**, strategic goals include creating an enabling environment [75,76,77,78,79].

## Framework on integrated people-centred health services current status in nine CEE countries (Table 1 – below)

**Table 1:** Framework on Integrated People-Centred Health Services: current status in 9 CEE countries.


POLICY OPTIONS AND INTERVENTIONS	COUNTRY 0 – DOES NOT EXIST; 1 – DEVELOPMENT PLANS; 2 – JUST BEING IMPLEMENTED; 3 – IMPLEMENTED								

	BELARUS	CZECH REPUBLIC	HUNGARY	LITHUANIA	POLAND	ROMANIA	RUSSIA	SLOVAKIA	UKRAINE

Strategy 1: Engaging and empowering people & communities (individuals and families, communities, informal carers, underserved and marginalized)									

Health education	1	2	2	2	2	2	2	1	2

Shared clinical decision making	1	1	1	1	1	0	1	0	2

Self-management	0	0	0	1	0	0	0	0	2

Community delivered care	3	1	2	3	3	3	3	0	2

Community health workers	1	2	1	1	0	3	1	0	1

Civil society, user and patient groups	1	3	2	2	2	3	2	2	2

Social participation in health	2	2	3	2	2	1	2	0	2

Training for informal carers	1	1	0	2	1	0	1	0	2

Peer support	0	0	0	0	0	0	0	0	2

Care for the carers	0	0	1	1	0	0	1	0	1

Equity goals into health sector objectives	0	1	1	1	1	2	0	0	2

Outreach programmes and services	0	1	1	1	1	1	1	1	2

Contracting out	1	2	1	2	2	0	2	0	2

Expansion of primary care	2	3	2	2	2	1	2	1	2

Strategy 2: Strengthening governance & accountability (bolstering participatory governance, Enhancing mutual accountability)									

Community participation in policy formulation and evaluation	1	2	0	1	1	1	1	0	1

National health plans promoting integrated people-centred health services	1	2	1	2	1	1	1	1	1

Donor harmonization and alignment with national health plans	1	1	1	1	1	1	1	0	1

Decentralization	0	1	1	1	1	2	1	0	2

Clinical governance	1	1	1	1	1	1	1	?	1

Health rights and entitlement	1	1	2	1	1	0	1		1

Provider report cards	0	0	1	0	0	0	0	?	0

Patient satisfaction surveys	2	3	2	3	3	3	2	3	3

Patient reported outcomes	1	1	1	1	1	0	1	0	1

Performance evaluation	0	1	1	2	1	1	1	3	1

Performance based financing and contracting	1	1	2	1	1	1	1	3	1

Population registration with accountable care providers	1	3	3	3	3	1	2	3	3

Strategy 3: Reorienting the model of care (Defining service priorities based on life-course needs, respecting social preferences; Revaluing promotion, prevention and public health; Building strong primary care-based systems; Shifting towards more outpatient and ambulatory care; Innovating and incorporating new technologies)									

Local health needs assessment	0	0	0	2	0	0	1	0	1

Comprehensive package of services	1	1	2	1	1	1	1	1	1

Strategic purchasing	1	2	1	2	2	1	2	?	1

Gender and cultural sensitivity	0	1	0	1	0	0	0	0	1

Health technology assessment	2	3	2	3	3	0	2	3	1

Population risk stratification	1	2	2	1	1	1	1	3	2

Surveillance, research and control of risks and threats to public health	1	2	2	2	2	1	2	3	2

Public health regulation and enforcement	1	2	3	2	1	1	1	3	2

Primary care with family and community-based approach	1	2	2	2	2	0	2	1	2

Multidisciplinary teams	1	2	1	2	1	2	1	1	1

Home and nursing care	2	3	3	3	3	1	3	3	2

Repurposing secondary and tertiary hospitals for acute complex care only	1	2	0	1	1	0	1	1	2

Outpatient surgery and day hospital	1	1	3	2	1	1	1	3	1

Shared electronic medical record	0	1	2	2	1	3	1	2	1

eHealth	0	1	2	2	1	3	1	2	2

Strategy 4: Coordinating services within and across sectors (Coordinating care for individuals; Coordinating health programmes and providers; Coordinating across sectors)									

Care pathways	1	1	1	1	1	1	1	0	2

Referral and counter-referral systems	2	3	3	3	3	3	2	3	2

Case management	0	0	0	2	0	2	0	0	1

Care transition	0	0	0	1	0	0	0	0	

Team-based care	1	2	1	2	1	1	1	0	1

Regional/district-based health service delivery networks	1	2	1	2	2	0	2	0	2

Integration of vertical programmes into national health system	1	1	2	1	1	0	1	1	1

Incentives for care coordination	1	1	1	2	1	0	1	0	0

Health in all policies	1	1	1	2	1	0	1	1	1

Intersectoral partnerships	1	1	1	2	1	1	1	1	1

Merging of health sector and social services	1	1	1	2	1	0	1	1	1

Integration of traditional medicine into health services	1	0	0	1	0	0	1	0	0

Coordinating preparedness and response to health crises	1	1	1	1	1	2	1	0	0

Strategy 5: Creating an enabling environment (Strengthening leadership and management for change; Strengthening information systems and knowledge; Striving for quality improvement and safety; Reorienting the health workforce; Aligning regulatory frameworks; Improving funding and reforming payment systems)									

Transformational and distributed leadership	1	1	1	1	1	0	1	0	1

Change management strategies	1	1	1	1	1	0	1	0	1

Information systems	1	1	2	2	1	1	1	0	1

Systems research and knowledge management	1	1	1	2	1	1	1	0	1

Quality assurance	1	2	2	2	2	1	2	2	2

Culture of safety	1	1	1	2	1	1	1	2	1

Continuous quality improvement	1	2	2	2	2	1	2	2	2

Workforce training	1	1	1	2	1	3	1	3	3

Multi-disciplinary teams	1	2	1	2	1	2	1	0	1

Improvement of working conditions and compensation	1	1	1	2	1	2	1	1	1

Provider support groups	1	1	1	1	1	1	1	0	1

Alignment of regulatory framework	1	1	1	1	1	1	1	1	1

Sufficient health system financing	1	1	1	1	1	1	1	1	1

Mixed payment models based on capitation	1	2	1	1	1	1	1	3	2

Bundled payments	1	1	1	1	1	1	1	?	2


Strategy 1. Engaging and empowering people & communities (individuals and families, communities, informal carers, underserved and marginalised). In all nine ECC countries, only self-management and civil society are implemented. For the most part, this strategy is under development. In the worst condition is self-management, which still does not exist.

Strategy 2. Strengthening governance & accountability (bolstering participatory governance, enhancing mutual accountability) either do not exist or are under development. Implemented strategies can be seen only in field patient satisfaction surveys and population registration with accountable care providers.

Strategy 3. Reorienting the model of care (defining service priorities based on life-course needs, respecting social preferences; revaluing promotion, prevention, and public health; Building strong primary care-based systems; shifting towards more outpatient and ambulatory care; innovating and incorporating new technologies) is implemented in the field of health technology assessment. Gender and cultural sensitivity and repurposing secondary and tertiary hospitals for acute complex care still do not exist.

In the field of strategy 4 – Coordinating services within and across sectors (coordinating care for individuals; coordinating health programmes and providers; coordinating across sectors), only referral and counter-referral systems are implemented. The biggest part of this strategy is still under development or, like care transition, does not exist.

Strategy 5. Creating an enabling environment (strengthening leadership and management for change; strengthening information systems and knowledge; striving for quality improvement and safety; reorienting the health workforce; aligning regulatory frameworks; improving funding and reforming payment systems) is still under development. Only part of workforce training was implemented in nine ECC countries.

## Conclusions

Coordinated people-centred care is crucial in care systems achieving the following goals of improving the general health of the population, improving individuals’ quality of care, and reducing per capita costs [80]. It should be kept in mind, however, that during the systemic transformation in the countries of Central and Eastern Europe, health care systems have undergone significant changes adjusting to the demands of a modern health industry leading to greater autonomy of institutions and professions, expansion of regional and local management, and a decision-making process which must account for the greater role of the health care market. As a result, these countries face major challenges related to coordination, system disintegration and a lack of control over the market environment [81]. Furthermore, given the growing health needs of ageing populations and epidemiological patterns, these countries need to increase funding, implement best practice reforms and improve cost-effectiveness while simultaneously integrating sectors through managed care and developing information and analytical knowledge through technological reforms to ensure management clarity [3].
